# Overlapping Features in Kawasaki Disease-Related Arthritis and Systemic-Onset Juvenile Idiopathic Arthritis: A Nationwide Study in Japan

**DOI:** 10.3389/fped.2021.597458

**Published:** 2021-07-20

**Authors:** Hikaru Kanemasa, Etsuro Nanishi, Hidetoshi Takada, Masataka Ishimura, Hisanori Nishio, Satoshi Honjo, Hiroshi Masuda, Noriko Nagai, Takahiro Nishihara, Tohru Ishii, Takenori Adachi, Satoshi Hara, Lisheng Lin, Yoshie Tomita, Junji Kamizono, Osamu Komiyama, Urara Kohdera, Saori Tanabe, Atsuo Sato, Shinya Hida, Mayumi Yashiro, Nobuko Makino, Yosikazu Nakamura, Toshiro Hara, Shouichi Ohga

**Affiliations:** ^1^Department of Pediatrics, Graduate School of Medical Sciences, Kyushu University, Fukuoka, Japan; ^2^Department of Perinatal and Pediatric Medicine, Graduate School of Medical Sciences, Kyushu University, Fukuoka, Japan; ^3^Department of Child Health, Faculty of Medicine, University of Tsukuba, Tsukuba, Japan; ^4^Department of Pediatrics, National Hospital Organization Fukuoka National Hospital, Fukuoka, Japan; ^5^Department of General Pediatrics and Interdisciplinary Medicine, National Center for Child Health and Development, Tokyo, Japan; ^6^Department of Pediatrics, Okazaki City Hospital, Okazaki, Japan; ^7^Department of Pediatrics, Japanese Red Cross Kumamoto Hospital, Kumamoto, Japan; ^8^Department of Pediatrics, National Hospital Organization Tochigi Medical Center, Utsunomiya, Japan; ^9^Department of Pediatrics, Tosei General Hospital, Seto, Japan; ^10^Department of Pediatrics, Juntendo University Urayasu Hospital, Urayasu, Japan; ^11^Department of Pediatrics, Kitakyusyu Municipal Yahata Hospital, Kitakyushu, Japan; ^12^Department of Pediatrics, National Hospital Organization Tokyo Medical Center, Tokyo, Japan; ^13^Department of Pediatrics, Nakano Children's Hospital, Osaka, Japan; ^14^Department of Pediatrics, Nihonkai General Hospital, Sakata, Japan; ^15^Department of Pediatrics, Yokohama Rosai Hospital, Yokohama, Japan; ^16^Department of Pediatrics, Osaka Red Cross Hospital, Osaka, Japan; ^17^Department of Public Health, Jichi Medical University, Shimotsuke, Japan; ^18^Fukuoka Children's Hospital, Fukuoka, Japan

**Keywords:** Kawasaki disease, juvenile idiopathic arthiritis, arthritis, immunosuppressive therapy, biologics

## Abstract

**Background:** Arthritis may occur after the diagnosis of Kawasaki disease (KD). Most cases are self-limiting; however, some patients require prolonged treatment.

**Method:** To characterize KD-related arthritis, 14 patients who required arthritis treatment within 30 days after the diagnosis of KD were recruited from the 23rd KD survey in Japan. Twenty-six additional patients were included from our tertiary center and literature review cohorts.

**Results:** The estimated prevalence of KD-related arthritis in Japan was 48 per 100,000 KD patients. Patients with KD-related arthritis had an older age at onset (52 vs. 28 months, *P* = 0.002) and higher rate of intravenous immunoglobulin (IVIG) resistance in comparison to those without arthritis (86 vs. 17%, *P* < 0.001). Among 40 patients, 18 had arthritis in the acute phase KD (continued fever-onset type) and 22 did in the convalescent phase (interval fever-onset type). Both showed a similar rate of complete KD or IVIG response. Interval-type patients required biologics for arthritis control less frequently (5 vs. 39%, *P* = 0.02) and had a higher 2-year off-treatment rate (100 vs. 43%, *P* = 0.009) than continued-type ones. Interval-types showed lower serum ferritin and interleukin-18 levels than continued-types. When continued-types were grouped according to whether or not they required biologics (*n* = 7 and *n* = 11, respectively), the former subgroup had higher ferritin and interleukin-18 levels (*P* = 0.01 and 0.02, respectively). A canonical discriminant analysis differentiated interval-type from continued-type with the combination of age, time to arthritis, and the ferritin and matrix metalloproteinase-3 levels.

**Conclusion:** Arthritis requiring treatment is a rare complication of KD. KD-associated arthritis includes interval-type (KD-reactive) and continued-type (true systemic-onset juvenile idiopathic arthritis [JIA] requiring biologics), and overlapping arthritis, suggesting the pathophysiological continuity of autoinflammation between KD and JIA.

## Introduction

Kawasaki disease (KD) is an acute febrile vasculitis of unknown etiology that primarily occurs in infants and children (1). The systemic vasculitis affects small- and medium-sized arteries, predominantly the coronary arteries. The advent of intravenous immunoglobulin (IVIG) reduced the incidence of coronary artery abnormalities (CAAs) in patients from 20 to 25% to <5% (1). However, KD remains the leading cause of acquired cardiovascular disease in children in developed countries. The incidence of KD is highest in Asian populations, especially Japanese. No single genetic or pathogenic factor or environmental substance has been identified as a cause of KD. The presentation of KD starts with high fever, followed by the sequential development of cervical lymphadenopathy, non-purulent conjunctivitis, and polymorphic exanthema. On the other hand, extra-cardiac manifestations often occur, including aseptic meningitis, anterior uveitis, and arthritis of small joints during the course of illness (2). Although KD may arise from innate immune activation in genetically predisposed individuals (3–5), the pathogenesis and treatment of non-cardiovascular complications remain to be explained.

Arthritis is a complication of KD that occurred in up to one-third of patients during the pre-IVIG era (1). It is generally recognized as a self-limited and non-deforming condition that is not associated with the destruction of articular cartilage (6), whereas several cases have reportedly developed severe arthritis resulting in the diagnosis of systemic-onset juvenile idiopathic arthritis (SoJIA) (7). SoJIA is classified as a subtype of juvenile idiopathic arthritis (JIA); it accounts for 10–20% of all cases of JIA (8). SoJIA patients present with continuous fever and then develop arthritis during the febrile or afebrile phase after treatment. Although the infection and immunological mechanisms are implicated in the onset and progression of JIA, the etiology remains unclear.

The diagnosis of SoJIA or KD at the disease onset is challenging because of common principal features and due to the absence of biomarkers for each disease (7, 9–13). In the case of arthritis, a considerable observation period is needed to determine whether the main lesion is joint or vascular inflammation. Despite the different response to IVIG, KD and SoJIA share the pathophysiology of inflammation during the disease course. There is little information about the occurrence, clinical characteristics, or treatment outcomes of arthritis complicated with KD. KD-related arthritis is classified into the early-onset and late-onset types, which develop within 10 days after the onset of KD and more than 10 days after the onset of KD, respectively (6). Patients with KD-related arthritis are more frequently complicated by CAAs than those without it (14). Patients with SoJIA or KD are at risk of macrophage activation syndrome (MAS). The early control of KD-related arthritis may therefore reduce the life-threatening complications. However, the differentiation of IVIG-resistant KD and SoJIA, which is important for indicating second-line treatment with disease-modifying drugs including biologics, corticosteroids, and other immunomodulation therapy, remains a dilemma for pediatricians. The timing of the onset of KD-related arthritis; early- or late-onset, does not predict the outcomes of arthritis treatment or exclude the diagnosis of *true* SoJIA at the time of step-up treatment.

In order to facilitate the optimal treatment of KD-related arthritis after the early exclusion of SoJIA, the present study aimed to investigate the prevalence, clinical expression, and treatment of arthritis by a recent nationwide survey of KD in Japan. Because of the insufficient number of cases, we further studied the treatment response of KD and KD-related arthritis after collecting additional cases from other cohorts in our tertiary center and from a literature review. The mode of onset of arthritis effectively discriminated between KD-reactive arthritis from SoJIA, and left a small set of borderline cases.

## Materials and Methods

### Ethics Approval and Consent to Participate

This study was conducted in accordance with the Declaration of Helsinki and the ethical guidelines for epidemiologic research of the Ministry of Health, Labor, and Welfare of Japan. Because of the retrospective study design, the requirement for written informed consent was waived. The study design was approved by the Institutional Review Board of Kyushu University (No. 28–94) and Jichi Medical University (No. 15–162).

### Diagnosis of KD

We used the diagnostic criteria of the Japanese KD Research Committee (14). Both complete and incomplete KD were included in the current study. Complete KD was defined by the presence of at least five of six principal symptoms, or the presence of four principal symptoms accompanied by CAAs. CAAs included dilations, aneurysms, stenosis, valvular heart disease, and myocardial infarction. Incomplete KD was diagnosed by the attending physicians. No KD-related arthritis was treated according to the same protocol that is used to treat JIA at the time of presentation.

### The Nationwide Survey of Patients Who Received the Treatment for Arthritis During the Course of KD

We conducted a nationwide study on patients who required active treatment for arthritis during the acute or subacute phase of KD (KD-related arthritis) as additional research of the 23rd nationwide survey of KD, which targeted patients diagnosed with KD from January 2013 to December 2014. Arthritis was determined by the presence of joint swelling, redness, and/or warmth, in addition to arthralgia. Otherwise, arthritis was confirmed by the signal change in the affected joints on magnetic resonance imaging. Patients with arthralgia alone, without other signs of arthritis were excluded from this study. The detailed methods of the survey have been described previously (15). Briefly, we sent questionnaires to all children's hospitals and institutions with ≥100 beds that had a pediatric department throughout Japan. The questionnaire included an initial questionnaire regarding the number of patients who required any treatment for arthritis within 30 days after the diagnosis of KD, as the definition of KD-related arthritis. The exclusion criteria were as follows: patients with dysmorphism or previously diagnosed disease. We then performed the secondary survey for each patient to determine the (1) background, (2) clinical characteristics and laboratory data at the time of the diagnosis of KD, (3) type of treatment of KD, (4) clinical and laboratory findings at the time of diagnosis of arthritis, (5) kind of treatment of arthritis and (6) outcomes. Arthritis treatments were classified into 5 categories: NSAIDs, corticosteroids, non-biologic immunosuppressants (methotrexate, cyclosporine A and tacrolimus), and biologic agents (infliximab, etanercept, canakinumab, and tocilizumab). Treatment outcomes were analyzed at 2 years after the diagnosis of KD.

### Chart and Literature Reviews of KD-Related Arthritis

In addition to the nationwide survey, we conducted another cohort analysis by chart and literature reviews as follows, because many of these rare cases did not include detailed information. We retrospectively investigated all patients with KD-related arthritis, as defined above, who received treatment at Kyushu University Hospital from 2007 to 2019. We further included all reported cases of KD-related arthritis by a review of the literature. Using the terms of “KD” and “arthritis,” we searched the PubMed database in June 2020 to identify publications describing these cases. The demographics, clinical characteristics, and all treatments were then extracted.

### Statistical Analyses

We used a chi-squared test to compare the proportions of categorical variables and the Mann-Whitney *U* test to compare the medians of continuous variables between the two groups. Bonferroni adjustment was used for multiple comparisons. For the analysis of long-term outcome, we included 19 patients only from the nationwide survey and our institution cohorts in order to dwarf possible difference in patient characteristics beyond statistical adjustments. A canonical discriminant analysis was performed to construct a prediction model, as we described previously (16). We included the months of age, days of illness at the onset of arthritis, and the serum ferritin and metalloproteinase-3 levels in a discriminant analysis. The Shapiro-Wilks test confirmed the normal distribution of these 4 variables. *P* values of <0.05 were considered to indicate statistical significance. The JMP Pro software program (ver. 14.2.0.; SAS Institute, Cary, NC, USA) was used for all of the statistical analyses.

## Results

### KD-Related Arthritis in the 23rd Nationwide Survey of Japan

**Figure 1:**
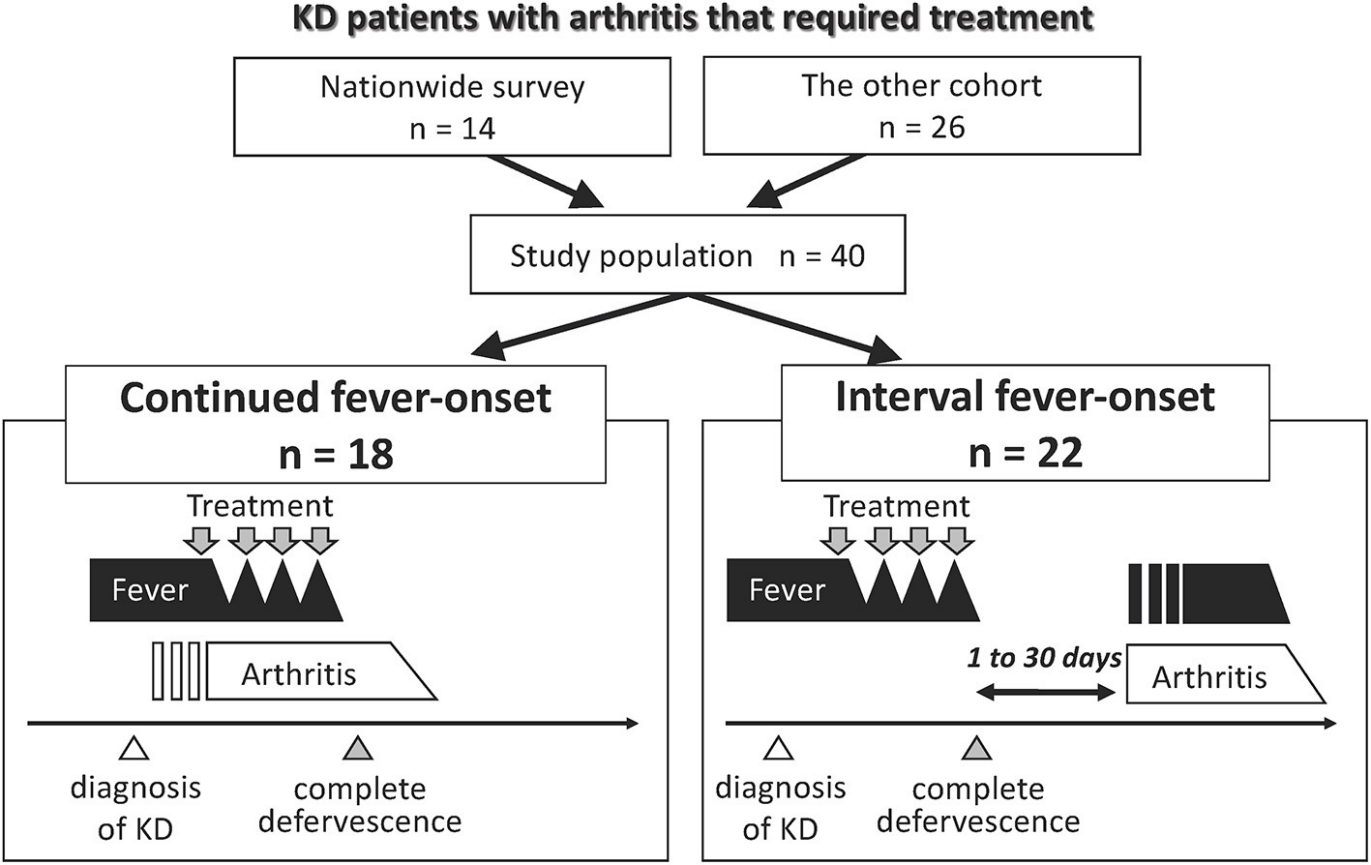
Group classification of patients with KD-related arthritis. Fourteen and 26 patients who suffered from arthritis that required treatment until 30 days following the diagnosis of KD were extracted from the 23rd nationwide survey in Japan and the additional cohorts, respectively. We combined two cohorts and classified the patients into two groups according to the mode of onset of fever and arthritis. Open triangle represents the time of the diagnosis of KD. Gray triangle represents the time point of complete defervescence. Gray arrow indicates the point when patients received treatment for KD or arthritis.

A total of 1,456 of the 1,943 eligible institutions replied to the initial questionnaire about KD-related arthritis in the 23rd nationwide KD survey (response rate, 74.9%), in which 31,679 patients received a diagnosis of KD in 950 institutions (Supplementary Figure 1). We excluded 2,595 patients who had <3 principal symptoms of KD or who improved spontaneously. Among 29,084 KD patients, 15 patients developed arthritis that required additional treatment. One patient was excluded because of confirmation of a >6 month-interval between the onset of arthritis and KD; thus, the pathophysiological association was undetermined. Fourteen patients with KD-related arthritis were finally identified (Figure 1). No patients had any previous history of arthritis at the onset of KD. The estimated prevalence of KD-related arthritis was 48 (95% confidence interval: 26–81) per 100,000 KD patients.

### KD-Related Arthritis in the Other Cohort

Nine patients with KD-related arthritis who were managed in our institution in this decade were identified. One was excluded as the interval between the onset of arthritis and KD was >6 months. The literature review identified 18 cases with KD and then arthritis. We also identified other 24 patients with KD-related arthritis but excluded them because of the onset of arthritis was very late (>6 months; *n* = 6) or due to a lack of detailed clinical information (*n* = 18). A total of 26 patients were included in the other cohort (Figure 1). No cases in this cohort overlapped with those in the data of the 23rd nationwide survey.

### Comparison Between KD Patients With and Without Arthritis

The demographics and clinical characteristics of KD were compared among KD patients who did not develop arthritis that required treatment in the nationwide survey (cohort 1), patients with KD and arthritis that required treatment in the nationwide survey (cohort 2), and the other cohort (cohort 3) (Table 1). There was no significant difference in sex, days of illness at the time of the diagnosis of KD, or type of KD among the three cohorts. The age at the onset of KD in cohorts 2 and 3 was older than that in cohort 1 (median, 52 and 45 months of age, respectively, vs. 28 months of age, *P* = 0.002 and 0.008, respectively). The rates of resistance to initial IVIG in cohorts 2 and 3 were much higher than those seen in KD patients without arthritis that required treatment (86 and 69%, respectively, vs. 17%, *P* ≤ 0.001 and < 0.001, respectively). The rate at which additional treatments for KD were required did not differ between cohorts 2 and 3.

**Table 1:** Demographic characteristics of KD patients with and without arthritis that required treatment.

	**1**	**2**	**3**	***P***		
	**KD without arthritis that required treatment, nationwide survey**	**KD with arthritis that required treatment, nationwide survey**	**KD with arthritis that required treatment, additional cohort**	**1 vs. 2**	**1 vs. 3**	**2 vs. 3**
Number of patients	29,069	14	26			
Months of age	28, 0–279	52, 20–87	45, 3–164	0.002	0.008	0.23
Male	16,658 (57%)	7 (50%)	13 (50%)	1.0	0.55	1.0
Days of illness at diagnosis of KD	5, 1–27	5, 3–9	5, 3–17	0.75	0.47	1.0
Complete KD	24,560 (85%)	11 (79%)	18 (69%)	1.0	0.10	0.72
1st IVIG resistance	4,986 (17%)	12 (86%)	18 (69%)	<0.001	<0.001	0.45
**Additional treatment**						
Repeated IVIG	5,573 (19%)	13 (93%)	19 (73%)	<0.001	<0.001	0.22
Infliximab	295 (1%)	2 (14%)	5 (19%)	0.026	<0.001	1.0
Plasma exchange	149 (0.5%)	1 (7%)	1 (4%)	0.21	0.13	1.0

*Data are shown as the number (%) or median and range. Bonferroni adjustment was used for multiple comparisons. KD, Kawasaki disease; JIA, juvenile idiopathic arthritis; IVIG, intravenous immunoglobulin*.

### Arthritis Developing in the Acute or Convalescent Phases of KD

There was no death due to CAAs or macrophage activation syndrome (MAS). We focused on two dissimilar courses of KD-related arthritis: the development of arthritis from the start of KD treatment to 24 h after complete defervescence was classified as continued fever-onset type, while the development of arthritis during the period from 1 to 30 days after complete defervescence was defined as the interval fever-onset type. According to these definitions, 18 and 22 patients were classified into the continued fever-onset and interval fever-onset groups, respectively. Complete defervescence was defined as a maximum body temperature of <37.5°C for 24 consecutive hours. The clinical laboratory findings and treatment outcomes of patients with the continued fever and interval fever types are summarized in Tables 2, 3, respectively. Despite the high rate of resistance to initial IVIG in both groups, the additional treatment of KD was only effective for 8 of 18 (44%) patients with the continued fever type, but was effective for all 22 patients with the interval fever type (Table 4). Continued fever-onset patients developed MAS more frequently (57 vs. 7%, *P* = 0.03) during the treatment course of arthritis. The serum matrix metalloproteinase-3 (MMP-3) levels in continued fever-onset cases were lower than those in the interval fever-onset cases (median, 45 vs. 160 ng/mL, *P* = 0.02). On the other hand, the serum ferritin level in the continued fever type was higher than that seen in the interval fever type (median, 3,163 ng/mL vs. 85 ng/mL, *P* < 0.001) The interleukin-18 (IL-18) levels in continued fever-onset patients were higher than those in interval fever-onset patients; however, the difference did not reach the statistical significance (51,500 vs. 711 pg/mL, *P* = 0.051). There was no difference in the numbers, locations, or symmetricity of the affected joints, or in autoantibody positivity. In both groups arthritis frequently involved the large joints of the lower limbs (knee 86 vs. 88%, hip 27 vs. 44%, and ankle 55 vs. 31%) and the small joints of upper limbs (wrist 60 vs. 43% and finger 42 vs. 50%). No patients were positive for rheumatoid factor or had an antinuclear antibody titer of >1:80. For the control of arthritis, 36% of the interval fever-type patients required NSAIDs alone (*P* = 0.03). In contrast, continued fever-onset patients required biologic agents more frequently in comparison to interval fever-onset patients (39 vs. 5%, *P* = 0.02). Focusing on the patients from the two cohorts other than the literature review cohort, 4 of 7 (57%) continued fever-onset patients were treatment-dependent at 2 years after the onset of arthritis, but all of the 12 interval fever-onset patients were weaned from therapy (*P* = 0.009).

**Table 2:** Detailed information of the continued fever-onset (*Co*.) patients.

**#**	**Gr**.	**Age, y.o**	**Sex**	**Type of KD**	**CAAs**	**Days of illness**		**Laboratory data after the development of arthritis**			**Treatment for KD**		**Treatment for arthritis**		**References**
						**Dx. of KD**	**Onset of arthritis**	**MMP3, ng/mL**	**Ferritin, ng/mL**	**IL-18, pg/mL**	**Course**	**Effect**	**Acute phase**	**After 2 years**	
1	*Co*	<0.5	M	I	D	6	15, F	na	653	na	IVIG → IVIG → IVIG → CS	Yes	CS	na	(17)
2	*Co*	0.5–2	M	C	D	na	na, F	na	Na	na	IVIG → IVIG	No	NSAIDs, CS, MTX	na	(18)
3	*Co*	0.5–2	M	I	D	na	na, F	na	Na	na	IVIG → IVIG/CS	Yes	CS, MTX, ETN, CAN, TCZ	na	9
4	*Co*	2–4	M	C	na	4	11, F	na	Na	na	IVIG → IVIG/CS	Yes	NSAIDs, CS	na	6
5	*Co*	2–4	M	C	na	6	4, F	na	Na	na	IVIG → IVIG	Yes	NSAIDs, CS	na	6
6	*Co*	2–4	M	I	No	9	14, FM	15	64,859	51,500	IVIG → IVIG → IFX → IVIG	No	NSAIDs, CS, HDMP, CyA, TCZ	na	NWS
7	*Co*	2–4	F	C	No	3	4, F	116	495	1,128	IVIG → IVIG → CyA	Yes	CyA	na	11
8	*Co*	4–6	M	I	PB	9	12	na	19,740	132,000	IVIG/CS	Yes	CS	na	12
9	*Co*	4–6	M	I	na	17	8	na	Na	na	IVIG	Yes	NSAIDs	na	6
10	*Co*	4–6	F	C	No	6	6, F	23	313	865	IVIG → IVIG → IFX → IVIG	Yes	NSAIDs, CS	No	KU
11	*Co*	4–6	F	C	No	6	6, FM	32	7,204	136,000	IVIG/CS → IVIG → IVIG → IVIG	No	NSAIDs, CS, HDMP, CyA, TCZ, VP-16, PE	Yes	NWS
12	*Co*	6–8	F	C	D	na	na, F	na	Na	na	IVIG → IVIG	No	CS, MTX, IFX	na	(18)
13	*Co*	6–8	F	C	No	5	na, F	29	2,700	17,600	IVIG → IVIG	No	CS, HDMP	na	NWS
14	*Co*	6–8	F	C	No	9	16, FM	24	12,330	68,000	IVIG → IVIG → HDMP	No	CS, CyA, TCZ	Yes	NWS
15	*Co*	6–8	F	I	No	5	31, F	81	3,626	202,000	IVIG → IVIG → PE	No	CS, CyA, TCZ	Yes	NWS
16	*Co*	6–8	F	I	No	13	12, F	190	15,600	145,000	IVIG → IVIG → UTI	No	NSAIDs, MTX, TCZ	Yes	KU
17	*Co*	>8	M	C	No	3	8, F	135	752	586	IVIG → IVIG → IFX → PE	No	NSAIDs, MTX, CyA	No	KU
18	*Co*	>8	F	C	No	4	6, FM	58	718	495	IVIG → HDMP	No	NSAIDs, CS, HDMP, TAC	No	KU

*KD, Kawasaki disease; C, complete KD; I, incomplete KD; CAAs, coronary artery abnormalities; D, dilation; PB, perivascular brightness; Gr., group; Dx., diagnosis; F, fever; FM, fever and macrophage activation syndrome; MMP3, matrix metalloproteinase-3; IL-18, Interleukin-18; IVIG, intravenous immunoglobulin; CS, corticosteroids; IFX, infliximab; CyA, cyclosporine A; HDMP, high-dose methylprednisolone; UTI, ulinastatin; PE, plasma exchange; NSAIDs, non-steroidal anti-inflammatory drugs; MTX, methotrexate; ETN, etanercept; CAN, canakinumab; TCZ, tocilizumab; ANR, anakinra; VP-16, etoposide; TAC, tacrolimus; NWS, Nationwide survey; KU, Kyushu University; na, not available*.

**Table 3:** Detailed information of the interval fever-onset (*Int*.) patients.

**#**	**Gr**.	**Age, y.o**	**Sex**	**Type of KD**	**CAAs**	**Days of illness**		**Laboratory data after the development of arthritis**			**Treatment for KD**		**Treatment for arthritis**		**References**
						**Dx. Of KD**	**Onset of arthritis**	**MMP3, ng/mL**	**Ferritin, ng/mL**	**IL-18, pg/mL**	**Course**	**Effect**	**Acute phase**	**After 2 years**	
19	*Int*	0.5–2	M	C	No	3	18, F	9	29	1,140	IVIG → IVIG/HDMP → CyA	Yes	NSAIDs, CS, CyA	No	NWS
20	*Int*	0.5–2	M	C	D	3	28, F	195	258	990	IVIG → IFX → IVIG → IFX	Yes	NSAIDs, CS, MTX	No	KU
21	*Int*	0.5–2	F	C	D	4	14, F	216	99	na	IVIG	Yes	NSAIDs	No	KU
22	*Int*	0.5–2	F	C	na	na	na	na	Na	na	IVIG → IVIG	Yes	NSAIDs, CS	na	(19)
23	*Int*	2–4	M	C	No	4	9, F	83	97	na	IVIG → IVIG/CS	Yes	NSAIDs, CS, MTX, CyA	No	NWS
24	*Int*	2–4	M	C	No	7	21, F	59	13	432	IVIG → IVIG/HDMP → CyA	Yes	NSAIDs, CS, CyA	No	NWS
25	*Int*	2–4	M	I	No	16	Na	na	Na	na	IVIG → IVIG → HDMP	Yes	CS, MTX	Yes	7
26	*Int*	2–4	F	C	No	4	6	181	67	na	IVIG	Yes	NSAIDs	No	KU
27	*Int*	2–4	F	C	No	5	26	na	71	na	IVIG/CS	Yes	NSAIDs, CS	No	NWS
28	*Int*	2–4	F	C	No	5	15, F	187	278	na	IVIG → IVIG → HDMP	Yes	NSAIDs, CS	No	NWS
29	*Int*	2–4	F	C	A	5	30, F	184	46	166	IVIG → IVIG → HDMP → IFX	Yes	NSAIDs, MTX	No	KU
30	*Int*	2–4	F	C	No	na	17, F	279	Na	na	IVIG → IVIG	Yes	NSAIDs	na	(20)
31	*Int*	2–4	F	C	na	na	na	na	Na	na	IVIG	Yes	NSAIDs	na	(19)
32	*Int*	4–6	M	C	D	4	10, F	na	Na	na	IVIG	Yes	NSAIDs	na	(21)
33	*Int*	4–6	M	C	D	4	21, F	103	Na	na	IVIG → IVIG → IVIG/CS	Yes	CS	No	NWS
34	*Int*	4–6	M	C	No	4	28	139	Na	na	IVIG → IVIG	Yes	NSAIDs, CS	No	NWS
35	*Int*	4–6	M	C	No	5	23, F	244	72	na	IVIG → IVIG/CS	Yes	NSAIDs	No	NWS
36	*Int*	4–6	M	I	No	7	na, FM	na	8,506	na	IVIG → IVIG → IFX	Yes	CS, ANR	No	7
37	*Int*	4–6	F	C	No	5	14, F	118	Na	na	IVIG	Yes	NSAIDs	na	(20)
38	*Int*	6–8	F	I	D, T	15	29, F	na	Na	na	IVIG → IVIG → HDMP	Yes	NSAIDs, CS	na	(22)
39	*Int*	6–8	M	C	na	na	na	na	Na	na	IVIG → IVIG	Yes	NSAIDs	na	(19)
40	*Int*	6–8	F	I	No	7	20	39	3,242	na	IVIG → IVIG/CS → IFX	Yes	na	na	NWS

*KD, Kawasaki disease; C, complete KD; I, incomplete KD; CAAs, coronary artery abnormalities; A, aneurysm; D, dilation; T, thrombus formation; Gr.,group; Dx., diagnosis; F, fever; FM, fever and macrophage activation syndrome; MMP3, matrix metalloproteinase-3; IL-18, Interleukin-18; IVIG, intravenous immunoglobulin; HDMP, high-dose methylprednisolone; CyA, cyclosporine A; IFX, infliximab; NSAIDs, non-steroidal anti-inflammatory drugs; CS, corticosteroids; NWS, Nationwide survey; KU, Kyushu University; na, not available*.

**Table 4:** Clinical characteristics of registered patients with KD and arthritis that required treatment.

	**Continued fever-onset**	**Interval fever-onset**	***P***
Number of patients	18	22	
Months of age	60, 3–164	46, 9–86	0.14
Male	9/18 (50%)	11/22 (50%)	1.0
Days of illness at the diagnosis of KD	6, 3–17	5, 3–16	0.24
Days of illness at the onset of arthritis	10, 4–31	20, 6–30	0.003
Complete KD	11/18 (61%)	18/22 (82%)	0.17
**Complications**			
Coronary artery abnormalities	5/15 (33%)	6/19 (32%)	1.0
Macrophage activation syndrome	4/7 (57%)	1/14, (7%)	0.03
**Treatment response**			
Response to single IVIG	2/18 (11%)	9/22 (41%)	0.07
Response to additional KD therapy^a^	8/18 (44%)	22/22 (100%)	<0.001
**Laboratory data at onset of arthritis**			
Rheumatoid factor, %positive	0/9 (0%)	0/18 (0%)	-
Matrix metalloproteinase-3, ng/mL	45, 15–190	160, 9–279	0.02
Ferritin, ng/mL	3,163, 313–64,859	85, 13–8,506	<0.001
Interleukin-18, pg/mL	51,500, 495–202,000	711, 166–1,140	0.05
**Treatment for arthritis**			
NSAIDs alone	1/18 (6%)	8/22 (36%)	0.03
Corticosteroids	15/18 (83%)	12/21 (57%)	0.10
Non-biologic immunosuppressants^b^	11/18 (61%)	6/21 (29%)	0.06
Biologic agents^c^	7/18 (39%)	1/21 (5%)	0.02
Except for infliximab	6/18 (33%)	1/21 (5%)	0.04
Off treatment at 2 years' follow-up	3/7 (43%)	12/12 (100%)	0.009

*Data are shown as the number (%) or median and range*.

a *Additional KD therapy includes conventional or high-dose corticosteroids, infliximab, plasma exchange transfusion, and ulinastatin other than IVIG*.

b *Non-biologic immunosuppressants include methotrexate and cyclosporine A*.

c *Biologic agents include infliximab, etanercept, tocilizumab, and canakinumab. KD, Kawasaki disease; IVIG, intravenous immunoglobulin; NSAIDs, non-steroidal anti-inflammatory drugs*.

### Difference Between Continued Fever-Onset Patients Who Required Biologics and Those Who Did Not

**Figure 2:**
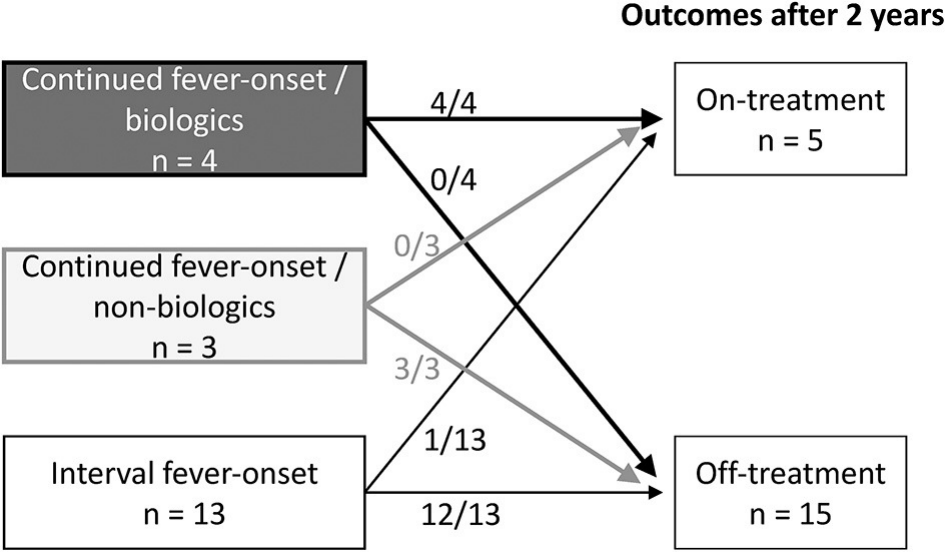
The treatment outcomes of three group-patients with KD-related arthritis. Information of the treatment outcomes of KD-related arthritis was available for 4 of 7 continued fever-onset patients /biologics, 3 of 11 continued fever-onset /non-biologics patients, and 13 of 22 interval fever-onset patients. All four continued fever-onset patients with arthritis that required biologic therapy and one interval fever-onset patient were receiving treatment at 2 years after the onset of disease. On the other hand, all three continued fever-onset patients who were managed without biologics and 12 of the 13 interval fever-onset patients were off treatment.

The above data indicated that continued fever-onset patients required long-term aggressive treatment for arthritis, whereas the arthritis of the interval fever-onset patients was relatively mild and transient. We then focused on the clinical difference between the continued fever-onset patients who required biologics (continued fever-onset/biologic, *n* = 11) and those recovered without biologics (continued fever-onset/non-biologic, *n* = 7) (Table 5). There was no significant difference in age, sex, duration until the diagnosis of KD and arthritis, type of KD, or the frequency of CAAs and MAS. On the other hand, the serum levels of ferritin and IL-18 were significantly higher in the continued fever-onset/biologic patients (median, 718 ng/mL vs. 12,330 ng/mL, *P* = 0.01; 997 pg/mL vs. 136,000 pg/mL, *P* = 0.02). All four patients in the continued fever-onset/biologics group were still receiving treatment for arthritis at the 2-year-follow-up examination, although all three patients in the continued fever-onset/non-biologic group had become medication-free (*P* = 0.03) (Figure 2).

**Table 5:** Clinical characteristics of the continued fever-onset patients who required biologics for arthritis control and those who did not.

	**Biologics not required**	**Biologics required**	***P***
Number of patients	11	7	
Months of age	60, 3–164	72, 10–87	0.59
Male	7/11 (63%)	2/7 (29%)	0.33
Days of illness at the diagnosis of KD	6, 3–17	9, 5–13	0.13
Days of illness at the onset of arthritis	8, 4–15	14, 6–31	0.06
Complete KD	8/11 (73%)	3/7 (43%)	0.33
**Complications**			
Coronary artery abnormalities	3/9 (33%)	2/9 (22%)	1.0
Macrophage activation syndrome	1/4 (25%)	3/3 (100%)	0.14
**Treatment response**			
Single IVIG	2/11 (18%)	0/7 (0%)	0.50
Additional KD therapy^a^	7/11 (63%)	1/7 (14%)	0.66
**Laboratory data at onset of arthritis**			
Matrix metalloproteinase-3, ng/mL	58, 23–135	32, 15–190	0.52
Ferritin, ng/mL	718, 313–19,740	12,330, 3,626–64,859	0.01
Interleukin-18, pg/mL	997, 495–132,000	136,000, 500–202,000	0.02
**Treatment for arthritis**			
NSAIDs alone	1/11 (9%)	0/7 (0%)	1.0
Corticosteroids	9/1 (82%)	6/7 (86%)	1.0
Non-biologic immunosuppressants^b^	4/11 (36%)	7/7 (100%)	0.01
Off treatment at 2 years' follow-up	3/3 (100%)	0/4 (0%)	0.03

*Data are shown as the number (%) or median and range*.

a *Additional KD therapy includes conventional or high-dose corticosteroids, infliximab, plasma exchange transfusion, and ulinastatin other than IVIG*.

b *Non-biologic immunosuppressants include methotrexate and cyclosporine A. KD, Kawasaki disease; IVIG, intravenous immunoglobulin; NSAIDs, non-steroidal anti-inflammatory drugs*.

### Three Types of KD-Arthritis Discriminated by the Prediction Model

**Figure 3:**
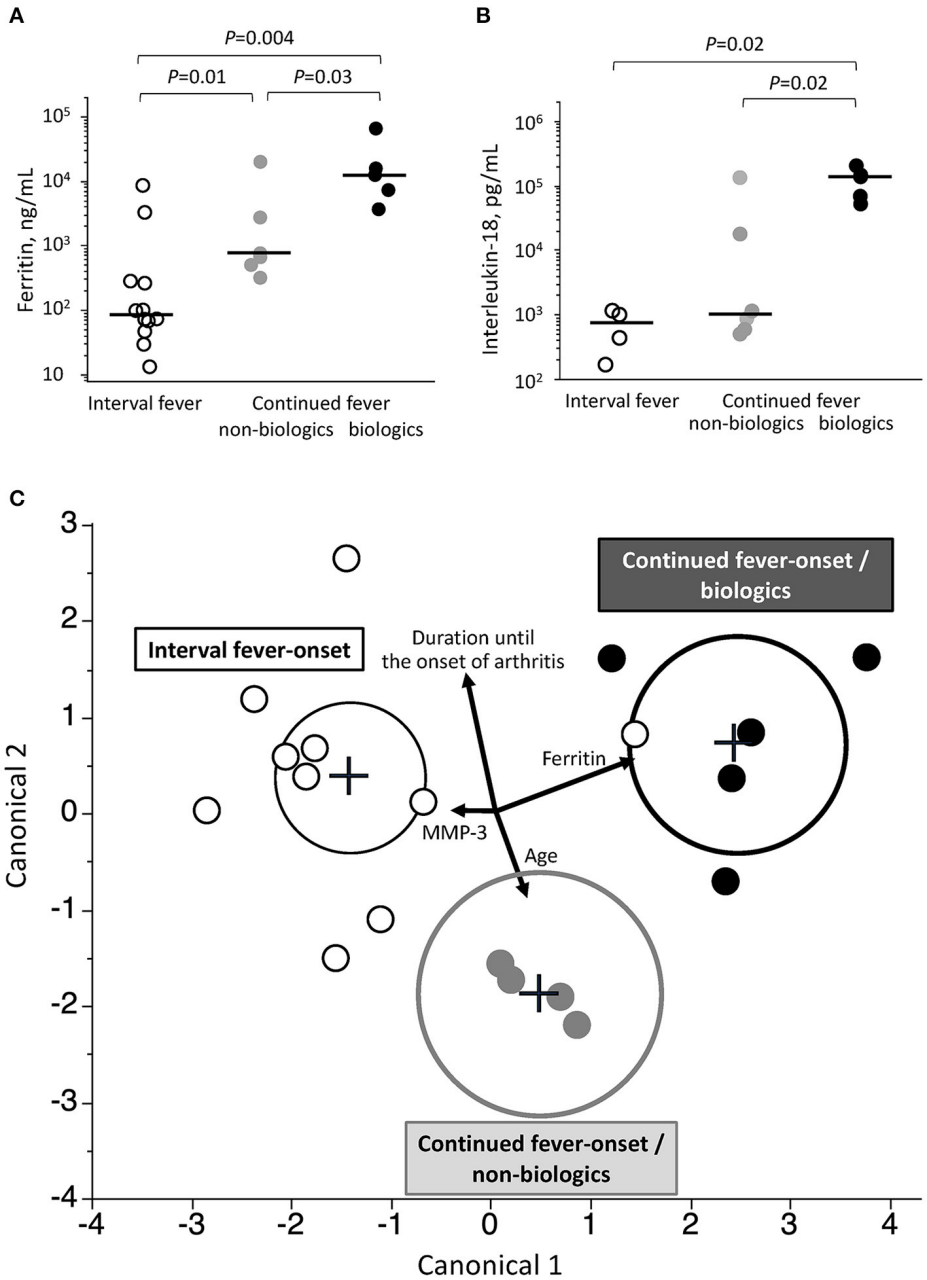
Associated clinical variables among the three groups of patients with KD-related arthritis. **(A,B)** Comparison of serum levels of ferritin **(A)** and interleukin-18 **(B)** at the onset of arthritis among patients in the continued fever-onset/biologic, continued fever-onset/non-biologic, and interval fever-onset groups. Black bars represent the median values. **(C)** A prediction model to predict the three types of the KD-related arthritis based on a canonical discriminant analysis. Each plot is determined using the combination of 4 clinical covariates at the onset of arthritis. Vectors indicate the direction and magnitude of each element. Black, gray, and white plots represent patients in the continued fever-onset/biologic, continued fever-onset/non-biologic, and interval fever-onset groups, respectively. The black, gray, and dotted circles in **(B)** denote the 95% confidence ellipse in the continued fever/biologic, continued fever-onset/non-biologic, and interval fever-onset types, respectively.

Among the five variables, including age, serum levels of ferritin, IL-18, and MMP-3 level, and days of illness at the onset of arthritis, only ferritin showed a significant difference among interval fever-onset, continued fever-onset/non-biologic, and continued fever-onset/biologic patients (Figures 3A,B and Supplementary Figure 2). We then focused on generating a prediction model that would be useful for facilitating an early diagnosis and intervention based on the abovementioned variables, with the exception of IL-18 due to the limited sample size. A canonical discriminant analysis effectively distinguished patients in the continued fever-onset/biologic, continued fever-onset/non-biologic, and interval fever-onset groups (Wilks' lambda 0.11, *P* ≤ 0.001) (Figure 3C). We applied log transformation to each variable for the transformation of non-Gaussian data into Gaussian data. The normality of the data for each variable was estimated by the Shapiro-Wilks test (*P* = 0.70, 0.25, 0.08, and 0.87, respectively). The scoring coefficients in the canonical plot were as follows: Canonical 1 = 0.90 Log [serum ferritin level, ng/mL]−0.18 Log [days of illness at the onset of arthritis]−0.19 Log [serum MMP-3 level, ng/mL] + 0.19 Log [months of age], Canonical 2 = 0.36 Log [serum ferritin level, ng/mL] + 0.91 Log [days of illness at the onset of arthritis]−0.02 Log [serum MMP-3 level, ng/mL]−0.56 Log [months of age]. However, this graph revealed that one interval fever-onset patient with complete KD (#40) had the properties of a continued fever-onset/biologic patient. This patient developed arthritis 8 days after the initial defervescence of KD, but showed a high serum level of ferritin at the time of the onset of arthritis. Unfortunately, no data were available for MMP-3, ferritin, or the IL-18 level in one interval fever-onset patient with methotrexate-dependent arthritis (#25) (Figure 2). The classification of 40 KD-arthritis patients into the early-onset and late-onset types did not make distinction between true SoJIA (after 2 years of treatment) and reactive arthritis (treatment-free at 2 years) (data not shown).

## Discussion

The first nationwide survey of KD-related arthritis revealed that the prevalence of KD-related arthritis was 48 per 100,000 KD patients in Japan. KD patients with arthritis that required treatment showed higher resistance to initial IVIG than those without arthritis. KD-related arthritis had two dissimilar patterns of expression: the refractory type, which occurred in the acute febrile phase; and the curable type, which occurred in the convalescent phase. This indicates the different pathophysiology associated with the joint inflammation of the two types. Furthermore, among the refractory type, patients who finally required biologics had high levels of serum ferritin and IL-18 in the acute phase and often required aggressive treatment, whereas those who improved without biologics had only moderate levels of ferritin and IL-18. The serum levels of ferritin and IL-18 at the onset of arthritis may be a good predictive biomarker to distinguish among these three types of KD-related arthritis. The discriminant model using the clinical variables at the onset of arthritis may help to more accurately classify the borderline disease between cases of KD-related arthritis and SoJIA.

The characteristics of interval fever-onset arthritis also resembled those of reactive arthritis (ReA). ReA occurs at 1–4 weeks after bacterial infection or inflammatory bowel disease, predominantly affecting the large joints of the lower extremities and occasionally affecting the wrists and fingers (23). No autoantibodies are found to be associated with the development of ReA. It usually resolves within 6 months without recurrence. In this context, interval fever-onset arthritis could be defined as ReA after KD, or KD-reactive arthritis.

High IVIG resistance was a hallmark of KD-related arthritis, including the interval fever-onset type. Considering that prolonged systemic inflammation may trigger arthritis, KD and ReA may be categorized into the disease spectrum of auto-inflammation. Recently, innate immunity has been recognized as a key contributor to the development of both KD and ReA. In mouse models (4), nucleotide-binding oligomerization domain-containing protein 1 ligand induced KD-like coronary vasculitis. It has also been reported that persistent gastrointestinal or urogenital infection is the source of causative pathogen-associated molecular patterns and leads to the development of ReA in association with a toll-like receptor 2 pathway (24). In fact, several pathogens, such as *Yersinia, Chlamydia*, and *Mycoplasma*, have been reported to be associated with the development of both KD and ReA (5, 25, 26). *Yersinia*-induced reactive arthritis is also related to the development of vasculitis (27). Understanding the shared mechanism of autoinflammation in KD and ReA may elucidate the pathogenesis of these diseases.

With the exception of one patient (Patient #8), patients with continued fever-onset arthritis had sustained fever at the onset of arthritis during the treatment of KD. Continued fever-onset/biologic patients showed high serum levels of ferritin and IL-18 and frequently became chronic, which were characteristics of SoJIA (28, 29). There have been several case reports of SoJIA after receiving the diagnosis of KD (7, 10, 13). The early diagnosis of SoJIA is difficult because it starts with high fever, skin rash, and lymphadenopathy, which is not differentiated from KD, without apparent arthritis (8, 30). In this setting, the majority of continued fever-onset/biologic patients might otherwise be “true SoJIA,” although the first presentation fulfilled the diagnostic criteria of KD. CAA is one of the common and specific complications of KD, whereas some patients finally diagnosed as SoJIA also developed CAAs (Patients #1–3, 8, and 12) (Tables 2, 3). We therefore compared the characteristics of CAAs developed in continued-fever onset and interval-onset fever group and found that there was no significant difference in the characteristics of CAAs between these groups. Patients in the continued fever-onset/non-biologic group also developed arthritis during the acute febrile phase. However, they showed moderate levels of ferritin and IL-18, and had a transient and curable course, similar to ReA. These patients might have had ReA with more excessive inflammation than typical ReA or the overlapping condition of SoJIA and KD-related ReA. The present study showed that the serum levels of ferritin and IL-18 can be useful to objectively predict the treatment response of arthritis during the course of KD. Our discriminant model may effectively differentiate the ReA type (interval fever-onset), SoJIA mimicking-KD type (continued fever-onset/biologic), and the intermediate type (continued fever-onset/non-biologic). It indicates that the combination of clinical variables at the onset of arthritis may be useful for pediatricians to select treatment and predict the joint prognosis.

The present study should be carefully interpreted as follows. First, the prevalence of KD-related arthritis was lower than those of KD-associated arthritis in reported in previous single-center studies in the IVIG era (2–13% of KD patients) (6, 19, 31, 32). This is explained by the recruitment of subjects: patients who needed any treatment for arthritis, but not the mild or self-limited form of arthritis, thus the small number of cases with KD-related arthritis might not typify the disease entity. Second, this study was based on the nationwide study of KD, although a part of patients, especially continued-fever onset/biologic patients, might have SoJIA as discussed above. Differentiation between KD and SoJIA is often difficult because both diseases are diagnosed on the basis of clinical symptoms. Our results emphasize the importance of considering SoJIA in patient with refractory KD even if they fulfilled the diagnostic criteria of KD. Third, 2-year outcome was available only for 19 patients among 22 from the nationwide survey and our institution cohorts. It is crucial to test whether the long-term outcome in the present study can be corroborated in other studies, thus prospective registration of KD patients targeting at long-term prognosis of arthritis and related symptoms are needed for the complete cure of KD.

## Conclusions

We conducted the first nationwide survey of KD patients who developed arthritis that required treatment. These patients were classified into three groups: (1) SoJIA mimicking KD, (2) KD-related ReA, and (3) arthritis with overlapping features between SoJIA and ReA. The serum levels of ferritin and IL-18 or the combination of clinical covariates at the onset of arthritis may help us to predict the treatment response and prognosis of arthritis. Although continued fever-onset patients were more likely to be treatment-dependent at 2 years after the onset of arthritis as compared to interval fever-onset patients in the present analysis, further studies are needed for the conclusion validity on the long-term prognosis. These findings, although preliminary, are potentially pivotal for risk stratification and treatment selection for patients who develop arthritis during the course of KD.

## Data Availability Statement

The original contributions presented in the study are included in the article/Supplementary Material, further inquiries can be directed to the corresponding authors.

## Ethics Statement

The studies involving human participants were reviewed and approved by the Institutional Review Board of the Kyushu University. Written informed consent from the participants' legal guardian/next of kin was not required to participate in this study in accordance with the national legislation and the institutional requirements.

## Author Contributions

HK contributed to the protocol development, performed the statistical analyses, interpreted the data, and drafted the initial manuscript. EN contributed to the protocol development, interpreted the data, and drafted the initial manuscript. HT contributed to the protocol development and critically revised the manuscript. MI and HN assisted in the protocol development and reviewed the manuscript. SHo (a Master of Science in Epidemiology) contributed to the statistical analysis and reviewed the manuscript. HM, NN, TN, TI, TA, SHa, LL, YT, JK, OK, UK, ST, AS, and SHi collected the data and reviewed the manuscript. MY, NM, and YN managed the data collection and reviewed the manuscript. TH conceptualized and designed the study and reviewed the manuscript. SO supervised the study and critically revised the manuscript. All authors approved the final manuscript as submitted and agreed to be accountable for all aspects of the work.

## Conflict of Interest

The authors declare that the research was conducted in the absence of any commercial or financial relationships that could be construed as a potential conflict of interest.
