# Supplementary material for: Overlapping Features in Kawasaki Disease-Related Arthritis and Systemic-Onset Juvenile Idiopathic Arthritis: A Nationwide Study in Japan
Source: Front Pediatr. 2021 Jul 20;9:597458. doi: 10.3389/fped.2021.597458 (PMC8329333; doi:10.3389/fped.2021.597458)

**Supplementary Figure**

**Figure S1.** Patient flow chart of the 31,679 patients with KD in the nationwide survey

**Figure S2.** Comparison of months of age (A), duration until the onset of arthritis (B), and serum level of matrix metalloproteinase-3 (C) at the onset of arthritis among patients in three groups. The black bar represents the median value.





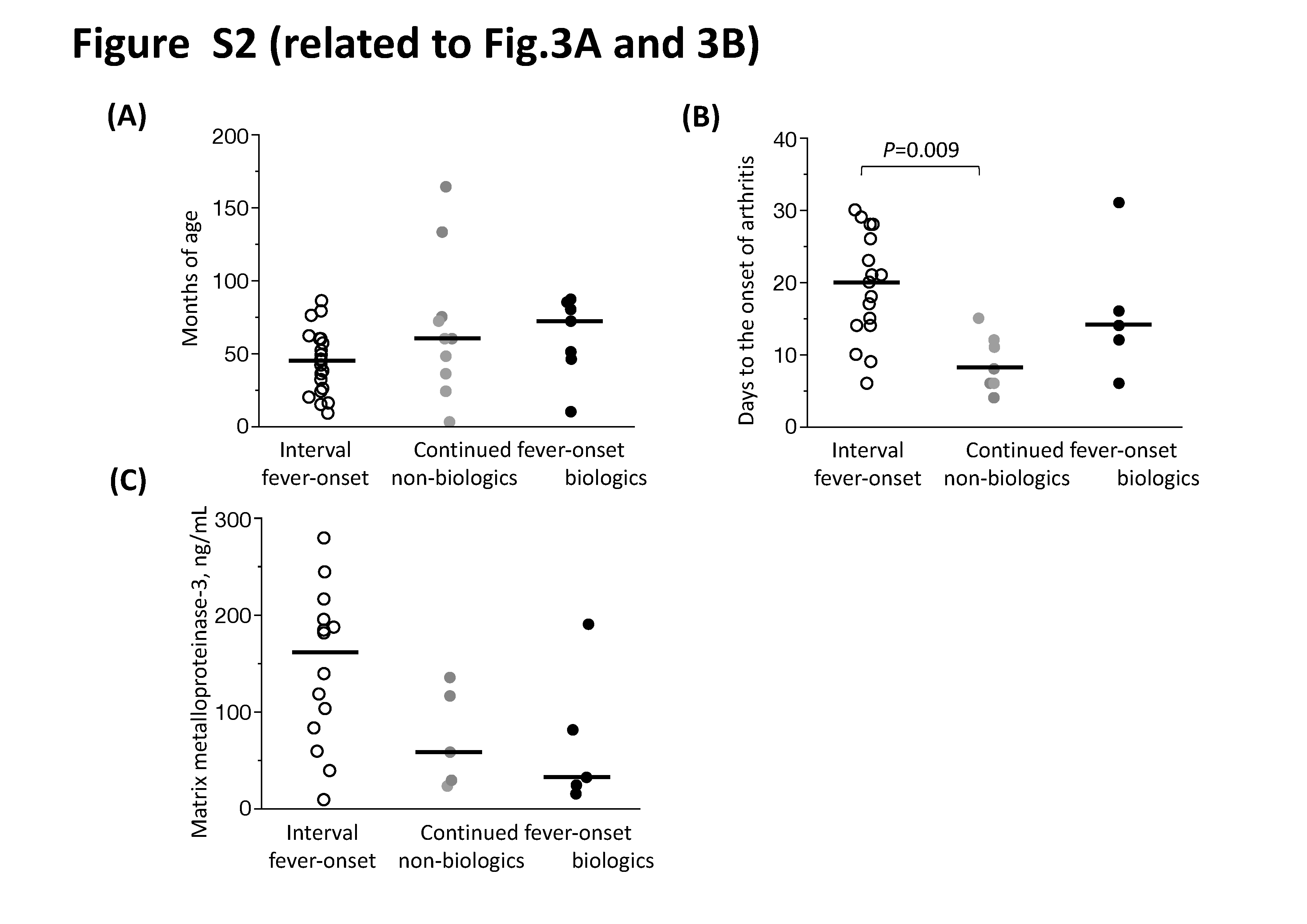

Supplement: Supplementary file 1 [file Data_Sheet_1.DOCX]
